# Trifluridine/tipiracil increases survival rates in peritoneal dissemination mouse models of human colorectal and gastric cancer

**DOI:** 10.3892/ol.2021.12772

**Published:** 2021-05-05

**Authors:** Norihiko Suzuki, Fumio Nakagawa, Teiji Takechi

Oncol Lett 14: 639-646, 2017; DOI: 10.3892/ol.2017.6258

Subsequently to the publication of the above paper, after having re-examined their raw data the authors have realized that Fig. 6 and Table II contained miscalculations. This figure and table appeared to show the results of one designated experiment, although they actually were intended to represent the results of the integration of two independent experiments. Specifically, one of the experiments evaluated the trifluridine/tipiracil (TFTD), tegafur, gimeracil and potassium oxonate (S-1) and cisplatin (CDDP) groups, whereas the other experiment evaluated the 5-fluorouracil (5FU) group alone. In addition, the description of the CDDP dosing schedule was also inaccurate: The CDDP dosing schedule on p. 640 of the Materials and methods section, right-hand column, “*In vivo antitumor activity*” subsection, should have been written as follows: “CDDP (7 mg/kg) was injected intravenously, once every **4** weeks, into the tail vein of mice (days **4** and **32**) for evaluating the effect on the gastric cancer MKN45 cell line” (essentially, the information reported for the weeks and days in this sentence was incorrect). Finally, the third sentence in the “*Antitumor activity of TFTD in the human gastric MKN45 intraperitoneal xenograft model*” subsection of the Results, towards the end of p. 642, should have read as follows (without the reference to 5FU): “TFTD exhibited a significant antitumor effect compared with S-1 and CDDP in the MKN45 intraperitoneal xenograft model (P<0.01; Table II).”

The revised versions of Fig. 6 and Table II, showing the corrected data without the 5FU group, are included on the next page. Note that the above errors did not affect the results or conclusions reported in this paper, and all the authors agree with this corrigendum. The authors thank the editor of *Oncology Letters* for presenting them with the opportunity to publish this Corrigendum and apologize to the editor and to the readership of the journal for any inconvenience caused.

## Figures and Tables

**Figure 6.**
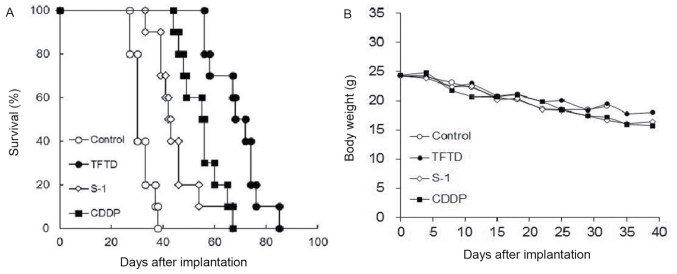
Effect of TFTD (A) on the survival of mice intraperitoneally transplanted with human gastric cancer MKN45 cells and (B) on the body weight of MKN45 tumor-bearing nude mice. Mice were treated with (○) vehicle, (●) TFTD (200 mg/kg, orally twice daily for 5 consecutive days followed by 2 drug-free days for 6 weeks), (◊) S-1 (10 mg/kg/day, orally once daily for 5 consecutive days followed by 2 drug-free days for 6 weeks) and (■) CDDP (7 mg/kg, intravenously once every 4 weeks for 6 weeks). Body weight was measured twice weekly. Results are presented as means (n=10). TFTD, trifluridine/tipiracil; S-1, tegafur, gimeracil and potassium oxonate; CDDP, cisplatin.

**Table II.** Antitumor activity of TFTD in the human gastric MKN45 intraperitoneal xenograft model.

	Survival day, median (ILS, %)			
	
Cell line	Control	TFTH	S-1	CDDP
MKN45	30 (−)	70^a–c^ (133)	43^a^ (42)	56^a^ (85)

ILS, increase in lifespan (%) = [(median survival time of treated group)/(median survival time of control group)-1]x100

a P<0.01 vs. control

b P<0.01 vs. S-1 group

c P<0.01 vs. CDDP group. TFTD, trifluridine/tipiracil; S-1, tegafur, gimeracil and potassium oxonate; CDDP, cisplatin.

